# The Aerosol Deposition Method: A Modified Aerosol Generation Unit to Improve Coating Quality

**DOI:** 10.3390/ma11091572

**Published:** 2018-09-01

**Authors:** Dominik Hanft, Philipp Glosse, Stefan Denneler, Thomas Berthold, Marijn Oomen, Sandra Kauffmann-Weiss, Frederik Weis, Wolfgang Häßler, Bernhard Holzapfel, Ralf Moos

**Affiliations:** 1 Department of Functional Materials, University of Bayreuth, 95447 Bayreuth, Germany; 2 Siemens AG, Corporate Technology, 81739 Munich, Germany; 3 Siemens AG, Corporate Technology, 91050 Erlangen, Germany; 4 Institute for Technical Physics, Karlsruhe Institute of Technology, 76021 Karlsruhe, Germany; 5 Palas GmbH, 76229 Karlsruhe, Germany; 6 Leibniz Institute for Solid State and Materials Research, 01171 Dresden, Germany

**Keywords:** aerosol deposition method (AD), aerosol generator, room temperature impact consolidation, brush generator, powder dispersion, magnesium diboride (MgB_2_)

## Abstract

Owing to its ability to produce dense thick-films at room temperature directly from a ceramic powder, the Aerosol Deposition Method (AD) possesses a unique feature in ceramics processing. For this technology, the aerosol generation of particles is a decisive part of reliable process control. However, there has only been a small amount of work published addressing this topic. In this work, we compare the aerosolization and deposition behavior of a fluidized bed generator with an aerosol generator with the rotary brush principle. While film properties very much depend on deposition time for the fluidized bed generator, films produced with the brush generator show a constant film profile, and their film thickness correlates with the controllable aerosol concentration and the duration of deposition. This type of aerosol generation may improve the setup towards a more reliable AD process.

## 1. Introduction

The importance of the aerosol generation in the Aerosol Deposition process is well known [1]. It is the key element in a reliable process and the main influencing factor in ensuring continuous progress towards industrialization. Few works, however, are dedicated specifically to this topic [1]; generally, schematic setups are presented [1,2,3,4,5,6]. In most cases, aerosol generators use the fluidized bed principle. Typically, the powder reservoir is vibrated and flushed by carrier gas to achieve a fluidized bed [6,7,8,9]. Particles and agglomerates of the powder are extracted into the aerosol state and transferred to the deposition chamber [5,9,10,11,12]. One has to take into account that the amount and state of the powder and the size of the distribution in the powder reservoir will change with time through continuous extraction. The composition of the aerosol with respect to particle size and distribution can be different to the bulk powder in the reservoir, since the aerosolization against gravity depends on the particle’s size and density. Small particles can be aerosolized easier than large particles or agglomerates.

In general, there are different ways and principles to aerosolize particles from powders. Several studies report on the dispersion of agglomerated particles in airflow [13,14]. The principles differ in the amount of dispersion energy applied to the particles [15]. Using dispersion from a fluidized bed, forces that separate particles are low compared to, e.g., impaction-related separating principles. It is suggested that agglomerates are either not deposited or hinder the deposition of homogeneous and dense nanostructured films, since parts of them are embedded in the film but do not fully consolidate by fracture and hammering to create a fresh, unsaturated surface that enables bonding [1,8,16,17]. Since the particle size in initial powders is usually between 100 nm and 10 µm, particles tend to agglomerate due to the uptake of moisture or through attraction by Van-der-Waals and tribo-electric forces. To prevent agglomeration or destroy agglomerates during aerosolization, and to achieve monodispersed particles, it is necessary to induce energy into aerosol by, e.g., gas shear flow and the impact of particles and agglomerates, etc., to overcome the adhesion energy and to separate the particles from each other [14,15]. Besides the separation of particles, the aerosol generator has to continuously dose a certain powder rate during the deposition process. This is also critical for the named particle size range. As one solution, this problem can be addressed by means of the granulation of relevant µm-particles to secondary particles [2,18,19], transferring them to a state of eased flowability.

In this study, we compare a fluidized bed generator of the kind commonly used in our and many other previous studies on aerosol deposition with a commercial aerosol generator, depositing sub-µm to µm magnesium diboride (MgB_2_) powders. MgB_2_ is a superconductor, and, furthermore, it has the advantage of good superconducting properties in particular as a polycrystalline, and non-textured film [20]. This technique could establish new and fast production methods for coated-tape superconductors in µm-thickness using the Aerosol Deposition (AD) Method. While electrical properties of films are discussed elsewhere [21,22,23], we investigate deposition behavior, running different experiments to compare the fluidized bed generator and a brush generator with respect to the predictability of film thickness.

## 2. Materials and Methods

**Figure 1:**
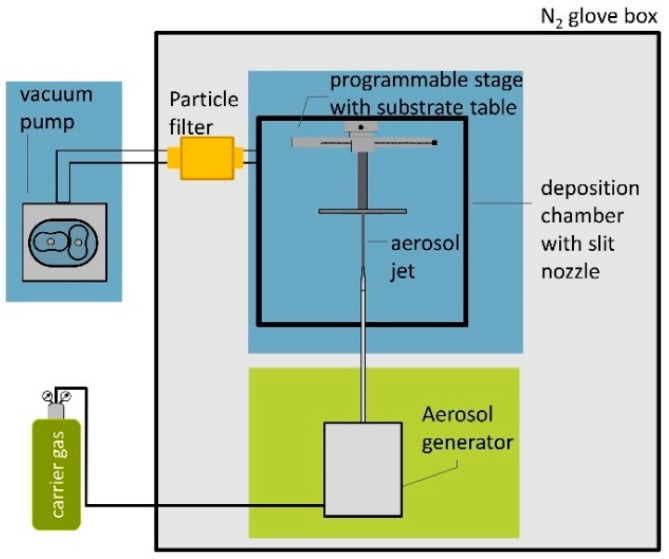
Schematic of AD device setup used for the brush generator experiments.

The MgB_2_ powder used in the experiments was supplied by Alfa Aesar. We used two different aerosol generator devices: A conventional fluidized bed generator (custom made) and, as second device, a commercial aerosol generator RBG 1000 SD from Palas GmbH, Karlsruhe, Germany. The AD apparatus and the working principle are described in detail in [9]. Figure 1 schematically describes the deposition device used for the studies with the commercial generator. Concerning the deposition with this brush-based system, the full AD device is kept in a N_2_-flushed glove box of reduced humidity (H_2_O < 50 ppm), due to the reactivity of the MgB_2_ powder with moisture [24]. For the conventional fluidized bed generator, a comparable device in ambient conditions was used. In order to guarantee reproducible dry and comparable powder condition for both experiments, the powder for the generator was filled in a glove box (Ar atmosphere).

**Figure 2:**
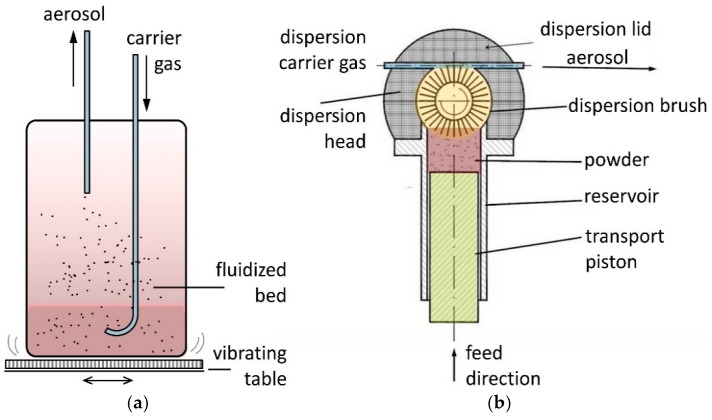
Working principle of (**a**) a fluidized bed generator and (**b**) of a powder dispersion unit with brush generator, modified after [25].

Furthermore, both aerosol generators were placed directly under the deposition chamber in order to ensure short tubing lengths. Figure 2 shows the working principles of the two generators. For the fluidized bed generator, the carrier gas (N_2_ 5.0 purity) is led into the powder filling, generating a fluidized bed (Figure 2a). The generator is fixed on a vibrating table. Parts of the aerosolized particles are extracted and delivered to the vacuum deposition chamber. The RBG 1000 SD is designed for low pressure and inert gas operation. The functional principle is shown in Figure 2b. For aerosol generation, the powder is filled into a reservoir. A homogeneous compaction of the powder filling improves the stability of the final aerosol concentration. The compacted powder is delivered upwards by a piston into a rotating dispersion brush (stainless steel or polymer). The piston is equipped with an additional fixture to the moving stage to prevent it from uncontrolled lifting due to the vacuum condition in the powder reservoir. The rotating brush is loaded with particles. They are dispersed from the bristles by a shear stream of carrier gas (N_2_ 5.0 purity). Compared with the fluidized bed principle, the mechanical treatment of the powder by the polymer rotating brush and the high velocity gas stream positively affect the de-agglomeration and particle separation.

The velocity of the piston movement is adjustable and can control the powder feed. Due to the setup and size of the piston, this is still a semi-batch-process. However, the process can be controlled better by the parameters piston velocity and brush rotation velocity, compared to the fluidized bed process. The carrier gas flow is set by a flowmeter. Films were deposited on glass object slides, as well as on 0.1 mm thick Hastelloy steel tape. Values for the deposition process are given in Table 1.

In the different experiments, we varied the piston velocity and the number of passes, while the remaining deposition parameters were kept constant. To obtain thicknesses and homogeneity, the films were analyzed by a laser-scanning-microscope Zeiss LSM 800 (Carl Zeiss AG, Oberkochen, Germany) and by a profile stylus instrument (Mahr Perthometer S2, Mahr GmbH, Göttingen, Germany). Besides thickness information (difference in height level from substrate to film surface), one can also gain information on surface roughness and morphology. SEM pictures for layer thickness determination and microstructural analysis from fracture cross sections were taken with SEM XL FEG, Philips GmbH, Amsterdam, The Netherlands.

## 3. Results

### 3.1. Film Thickness Evolvement for the Different Generator Principles

**Figure 3:**
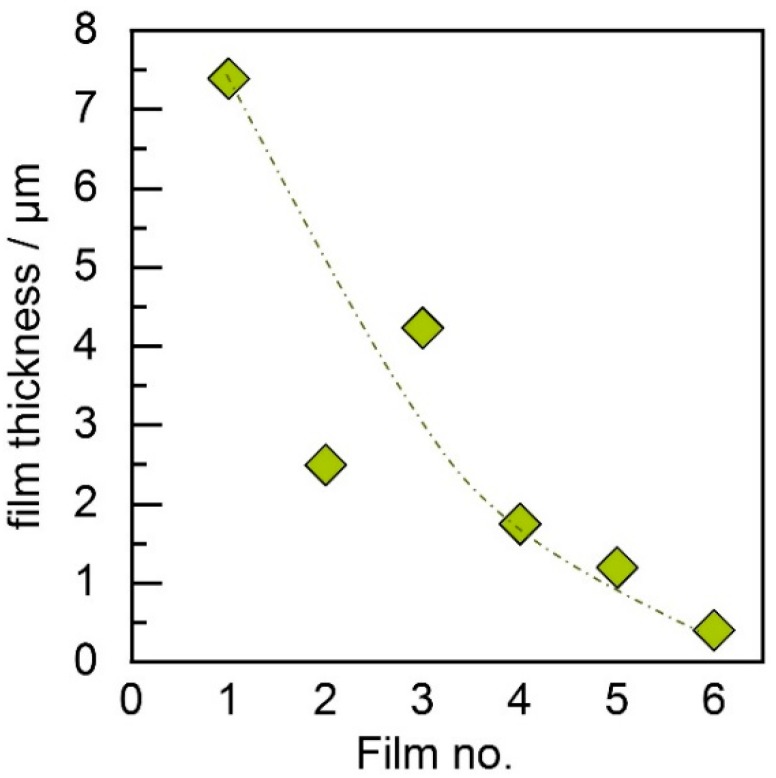
Development of film thickness over deposition time for the fluidized bed generator; the dotted line serves as guide for the eye.

The first experiments were intended to compare the two different aerosolization principles. With the conventional fluidized bed generator, six films were deposited subsequently on glass substrates with one batch of powder without refilling in order to record deposition behavior over time. The overall deposition time for all films was around 20 min. All other parameters were kept constant for comparable conditions between the films. With values for *d*_10_ of approx. 1.1 µm, *d*_50_ of 9.6 µm, and *d*_90_ of 51.2 µm, the MgB_2_ powder used here has quite a high number of coarse particles. Nevertheless, we achieved films of thicknesses up to 7 µm. The average film thickness over the film number is given in Figure 3.

As can be found for the fluidized bed generator, consecutively deposited films show a strong decline in thickness over the deposition time from approx. 7.5 µm for the first to 0.5 µm for the last film. Since deposition parameters remained constant, the change in film thickness is most likely related to a change in the aerosol, either in particle concentration or in quality.

**Figure 4:**
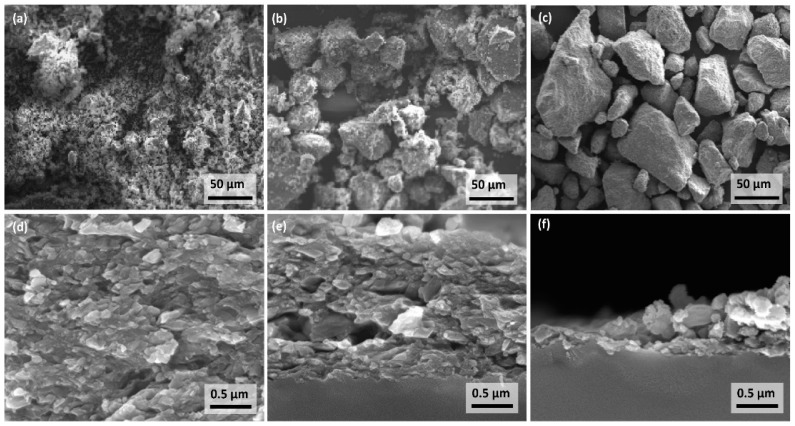
Development of powder agglomeration over deposition time for powder in the fluidized bed generator after runs (**a**) 1, (**b**) 4, and (**c**) 6, and corresponding fracture cross section of the respective film morphology for (**d**) 1, (**e**) 4, and (**f**) 6. Powders were probed from fluidized bed generator after each run.

To investigate the reason for the change in deposition behavior, the powder of the fluidized bed was probed and analyzed after certain runs. Figure 4a–c shows representative SEM micrographs of the powder morphology and Figure 4d–f the corresponding fracture cross section of the films. Due to the broad particle size distribution, the reason for the change in deposition behavior can be resolved clearly: The powder micrographs reveal that fine particles are extracted with time, leaving large particles and agglomerates in the reservoir. With this, the amount of powder and thus the aerosol concentration reduces continuously but also uncontrollably. This is also the case for powders with smaller particles and sharper size distribution. In the beginning of the process, most particles are bound in small and soft agglomerates that can be aerosolized much more easily by, e.g., shear flow. It is assumed that these agglomerates also increase in diameter over time through, e.g., triboelectric forces. The corresponding fracture cross sections of the films reflect the trend in Figure 3 and show the rough and inhomogeneous nature of the latter films. With time, less particles are aerosolized, leading to films of low thickness and reduced quality. The reduction of the deposition rate and a decrease of film quality with deposition time is a well-known aspect of fluidized bed generators, e.g., from [26,27]. However, this study provides a good contrast to our experiments below.

**Figure 5:**
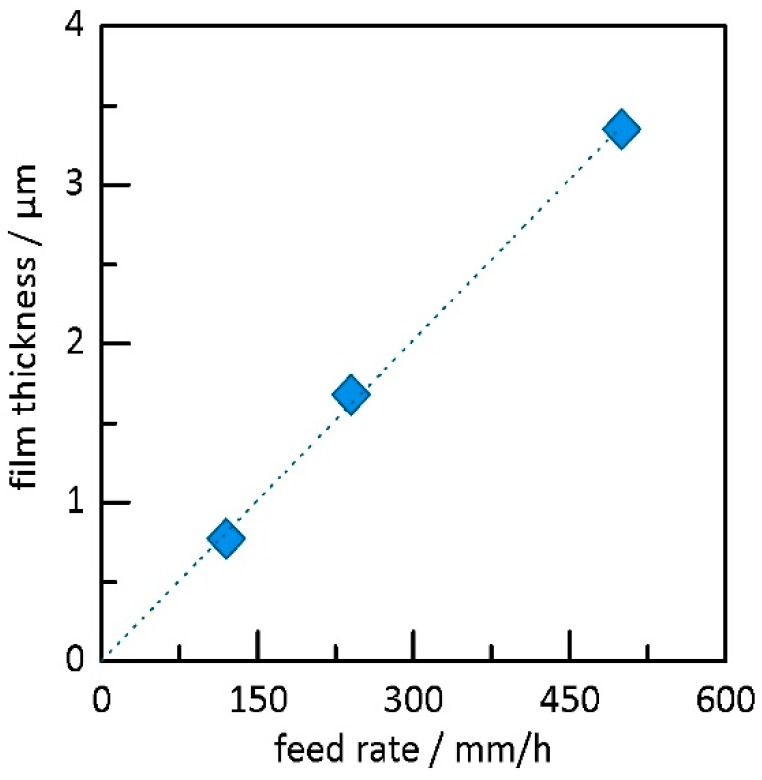
Development of film thickness over powder feed rate for the brush-based generator; the dotted line serves as guide for the eye.

In case of the brush generator, the powder feed rate was varied through the piston speed. With this experiment, the correlation between aerosol concentration, deposition rate, and film thickness was investigated. First experiments using the brush generator and same powder as mentioned before showed a lower layer quality and layer thicknesses of only around 1 µm. We think this is related to the higher gas flow and, as a consequence, to the higher kinetic energy of particles that might harm the film through an effect similar to sandblasting [17,28,29,30] induced by the bigger particles. Thus, a milling procedure for the powder has been applied. According to this, values for particle size could be decreased to *d*_10_ = 0.8 µm, d_50_ = 1.8 µm, and *d*_90_ = 4.5 µm, increasing the fraction of powder that can be deposited. The piston velocity was varied in three steps from 120 mm/h to 500 mm/h, which is equivalent to a powder feed rate of approximately 0.37–1.55 g/min or 0.02–0.08 g/L (standardized volume) when related to the carrier gas flow, which can serve as an estimate of the aerosol concentration. All other parameters remained constant, as shown in Table 1. Corresponding film thicknesses are shown in Figure 5. The film thickness increases linearly with increasing feed rate. Obviously, the aerosol concentration is linearly connected to the feed rate, which means that dispersion of particles works well for the feed rates used here. In other words, the adjustable piston velocity or the feed rate is a good measure of the deposition rate and the corresponding film thickness, which is in strong contrast to the fluidized bed system in which the aerosol concentration cannot be fixed, provoking time-dependent deposition behavior. However, the system seems to be more sensitive to powder particle size, likely due to the higher energy induced into the particles. Especially, large particles might hinder the film formation. More experiments are necessary to prove this theory.

Through the milling treatment of the powder, the deposition efficiency will be increased, meaning that the maximum values of film thickness will not be comparable between the two generator systems [31].

However, as we can conclude from the first comparative experiment, the change in powder distribution and morphology through uncontrolled extraction and continuous agglomeration reduce the available powder in the reservoir of the fluidized bed generator. This is undoubtedly related to this generator principle and leads to the changing deposition behavior. However, it is not clear which of both factors dominate the behavior. The shaking of the powder promotes surface charging leading to agglomeration, while the force induced by the shear flow of the gas in this kind of generator is insufficient for de-agglomeration. In contrast to the fluidized bed principle, the mechanical treatment by the brush in the dispersion head of the brush-based generator in combination with the shear flow (higher velocity through higher gas flow) seem to maintain constant dispersion [15]. Although the initial powders were different for the two generators experiments, the process principles can be compared with regard to predictability and continuity of deposition behavior.

### 3.2. Deposition Experiments with Aerosol Brush Generator

**Figure 6:**
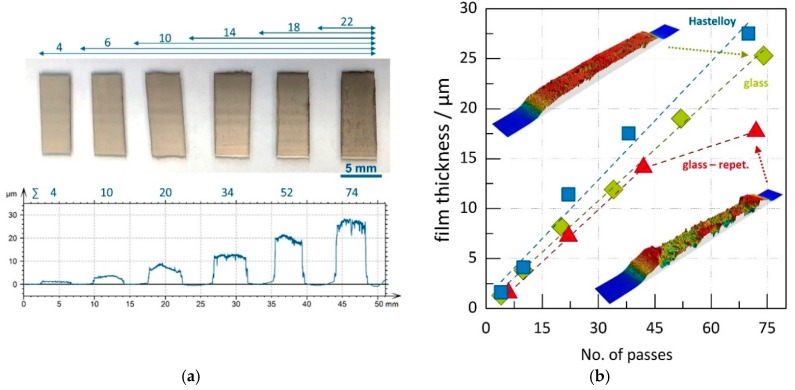
Variation of substrate passes: (**a**) image of films on glass substrate and corresponding profile and (**b**) film thickness vs number of passes for different substrates. Dotted lines serve as guide for the eye.

While in the first experiment the aerosol concentration was varied in a consecutive step, we investigated the number of passes at constant feed rate of 120 mm/h. This feed rate was chosen in order to have sufficient deposition time to vary the number of passes. Glass and Hastelloy served as substrates. An image of a coated glass substrate is given in Figure 6a. The substrate holder moves over the nozzle from left to right. The glass substrate was masked by sticky tape to clearly distinguish between the profiles of the six films. The total number of passes, as well as a profile measurement of the films, is given for each film under the image. The profile shows a continuously increasing thickness with increasing number of passes. The film thicknesses in Figure 6b are averaged values over the film width. Since the film thickness would be falsified by the substrate bending (originated from the film stress), profile curves were corrected by subtracting a polynomial function fitted to the substrate bending in the overspray area. As for the last experiment, we here see a linear relation between the number of passes and the films thickness. On the glass substrate (green diamonds), as well as on the Hastelloy (blue squares), film thicknesses of over 25 µm can be reached, giving a volumetric deposition rate of approximately 0.2 mm^3^/min. With respect to the strong substrate bending in case of the thin Hastelloy substrate, the film thickness measurement might involve more deviation. The results indicate that the principle of aerosol dosing used here offers a reliable method for a more predictable AD process.

For the 100 µm thin Hastelloy substrate, we observed strong substrate bending after deposition. With the mechanical properties and geometry of film and substrate, the film strain of the 11.4 µm and 17.5 µm thick films on Hastelloy can be approximated by the equations for thin films by Stoney [32], giving values for stresses of 0.42 and 0.36 GPa within the layer, respectively [12].

Concerning the repetition of the experiment on glass (red triangles), the linear correlation of the mean film thickness and the number of passes is valid up to 45 passes. It shows good agreement with the previous run. However, the film deposited with 72 passes shows a lower thickness. A closer look at the film profile reveals an inhomogeneous and rough surface (see insets Figure 6b).

**Figure 7:**
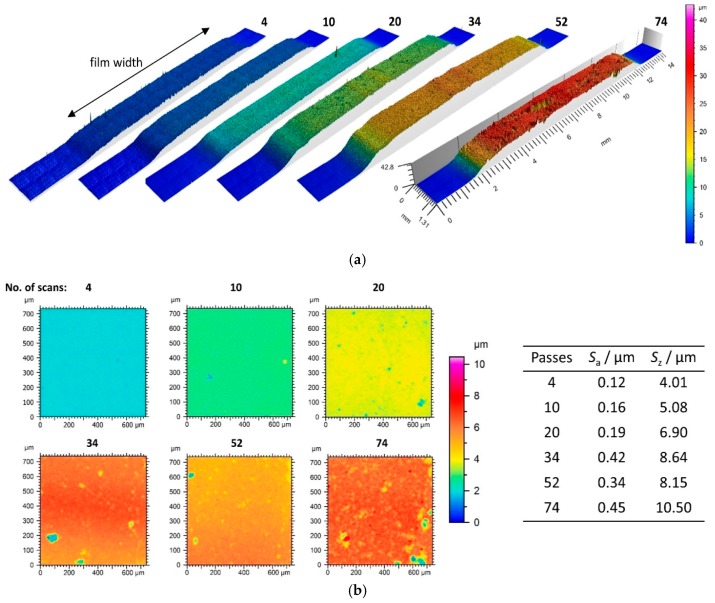
Pseudo-color areal surface profiles of MgB_2_ films on glass substrate with (**a**) profiles parallel to nozzle width and (**b**) top-view on film surface and corresponding surface roughness values *S*_a_ and *S*_z_. Numbers describe the number of passes for layer generation.

Three-dimensional width profiles of all films on glass (green diamonds) are given in Figure 7a with increasing number of passes from left to right. The film shows a homogeneous thickness over the width. One can see slight spalling and irregular craters in the profile of the thickest film. This is also reflected in the surface roughness values *S*_a_ (surface roughness—mean arithmetic height) and *S*_z_ (surface roughness—maximum height) derived from top-view of high resolution measurements (see Figure 7b). While the arithmetic roughness shows quite low values of *S*_a_ < 0.16 for low thicknesses, values of *S*_a_ increase significantly for thicker films.

As can be seen in the top-view surface images, one has to distinguish between increasing roughness and the occurrence of local surface defects in the film. While the thin films show just few and isolated flaws, the defects in the films with 34 to 74 passes become more frequent and more pronounced in size and depth. Nevertheless, even for high film thicknesses of 25 µm, the defects in the film are still mainly isolated and do not dominate the appearance of the surface. Flaws can be due to insufficient consolidation of particles or agglomerates that are embedded in the film. On the one hand, embedded flaws can act as predetermined breaking points and—in combination with high film strain—lead to laminar delamination, as shown in the inset in Figure 6b. As known from literature, the achievable film thickness reduces with increasing inclination angle of the substrate [33].

Although the here-shown setup uses plane substrate slopes, surface craters might have a similar effect on film growth rather leading to growth of these defects in depth and width with the growing film thickness. In order to prevent cratering and surface inhomogeneities, one has to address the consolidation of the film and prevent implementation of loose particles and agglomerates into the film, or even prevent them from entering the aerosol. The high shear forces in the rotating brush generator are particularly beneficial at removing larger agglomerates. However, some smaller agglomerates might still be present in the aerosol. Further aerosol processing with classification would be necessary to almost completely remove such agglomerates. Other effects that need to be considered are particle losses, deposition, and resuspension in the pipes connecting the aerosol generator and deposition chamber. Piping should be kept as short as possible with no sharp bends and diameter changes. Even though this was taken care of in the current setup and the effect might be small, it could not be completely avoided and must be kept in mind.

## 4. Conclusions

The results clearly show the influence of the aerosolization principle on the film quality. The results on the fluidized bed generator give an example of the changes appearing in the aerosol owing to continuous agglomeration and particle extraction with time. Particles and soft agglomerates are discharged with time, leaving large, undispersable particles and hard agglomerates. This changes the deposition behavior, reduces the deposition rate, and affects film quality negatively. Compared to that, AD process operated with a commercial brush disperser shows promising film quality, even for MgB_2_ films as thick as 25 µm. Films can be deposited on steel and on glass substrates, while, importantly, the thickness correlates linearly with the feed rate, aerosol concentration, and the deposition time, making the overall process adjustable, more predictable, and therefore more suitable for continuous operation. The improvements are thought to originate from better dispersion efficiency, which is caused by the higher carrier gas flow that induces higher shear forces to separate particles along with the improved control of the powder feed rate. However, we also found that the system is more sensitive to large particles, most likely due to the higher carrier gas flow and the resulting increase in kinetic energy, and possibly due to associated mechanisms such as sandblasting, since all kinds of particles (small to large) are discharged from the reservoir. This, in turn, affects the necessary pre-conditioning of the powder. Nevertheless, further studies have to be conducted to investigate long-term dispersion and deposition behavior. For this, brush-based systems with a bigger powder reservoir would be applicable. In addition, other materials should be deposited as well, to investigate the influence on morphological and functional properties of films compared to fluidized bed generators.

## Figures and Tables

**Table 1:** Parameters used in the deposition experiments with both different aerosol generation units.

Deposition Parameters	Fluidized Bed Generator	Brush Generator
Piston diameter/mm	-	20
Brush rotation/rpm	-	1200
Carrier gas	N_2_ 5.0 purity	
Flow rate/nL/min	6	20
Scan speed/mm/s	1.5	1
Piston speed/Feed rate/mm/h	-	120–500
Nozzle orifice/mm²	5	
